# Performance of breast cancer screening methods and modality among Chinese women: a report from a society-based breast screening program (SBSP) in Shanghai

**DOI:** 10.1186/2193-1801-2-276

**Published:** 2013-06-24

**Authors:** Miao Mo, Guang-yu Liu, Ying Zheng, Lian-fang Di, Ya-jie Ji, Li-lang Lv, Ying-yao Chen, Wei-jun Peng, Jie-ru Zhu, Ping-ping Bao, Jian-hui Ding, Cai Chang, Jian-feng Luo, Zhi-gang Cao, Wang-hong Xu, Zhi-min Shao

**Affiliations:** Shanghai Cancer Hospital, Fudan University, 270 Dong An Road, Shanghai, 200032 China; Department of Epidemiology, School of Public Health, Fudan University; Key Laboratory of Public Health Safety, Ministry of Education (Fudan University), 138 Yi Xue Yuan Road, Shanghai, 200032 China; Department of Oncology, Shanghai Medical College, Fudan University, 270 Dong An Road, Shanghai, 200032 China; Department of Cancer Prevention and Control, Shanghai Municipal Center for Disease Control and Prevention, 1380 Zhong Shan Xi Road, Shanghai, 200336 China; Community Health Care Center of Qibao County, Minhang District, 94 Fu Qiang Road, Shanghai, 201101 China

**Keywords:** Breast cancer screening, Clinical breast examination, Mammography, Breast ultrasonography, Sensitivity, Specificity

## Abstract

To evaluate the screening performance of individual and combined use of clinical breast examination, ultrasonography and mammography in Chinese women, we conducted a biennial breast cancer screening program among 14,464 women aged 35 to 74 years old who lived in Qibao County, Minhang district of Shanghai, China, between May 2008 and Sept 2012. All participants were submitted to clinical breast examination, and then women with positive results and all women at age of 45-69 years old were preformed breast ultrasonography and mammography. The examination results were compared against pathological findings as the gold standard of reference. A total of 66 women were diagnosed with breast cancer in the two rounds of the screening, yielding an incident rate of 194 per 100,000 person-years. The sensitivity of clinical breast examination, ultrasonography and mammography alone were 61.4%, 53.7% and 67.3%, respectively. While mammography performed better in elder age groups and hormone receptor positive disease groups, ultrasonography had a higher sensitivity in younger age group and did not differ in sensitivity by estrogen receptor or progesterone receptor status. Combined use of the two imaging examinations increased the sensitivity in almost all age groups, but had a higher sensitivity in hormone receptor positive cancers than in those negative. Our results suggest that the Qibao modality is an effective strategy for breast cancer screening among Chinese women, especially for early detection of elder and hormone receptor positive breast cancer.

**Electronic supplementary material:**

The online version of this article (doi:10.1186/2193-1801-2-276) contains supplementary material, which is available to authorized users.

## Introduction

Breast cancer is the most common malignancy among women around the world. It is estimated that, in 2008, 1.38 million women were newly-diagnosed with breast cancer and 458,000 died of the malignancy (Ferlay et al. 2010). With rapid socioeconomic development and great translation in lifestyles in China, breast cancer has been becoming one of the leading public health issues in the country. In Shanghai, the biggest city in China, the overall age-adjusted incidence increased by 134% between 1975 and 2004 (Shanghai Municipal Center for Disease Control and Prevention 2007).

So far, no effective approach has been developed to prevent the incidence of breast cancer. Therefore, early detection of the malignancy is of the most important to improve the life of quality, prolong the survival of patients and prevent the premature death from the disease. During past decades, while both incidence and mortality of breast cancer have been rising in China and other Asian countries (Shin et al. 2010), the mortality of the disease has decreased in the West although the incidence also has been increasing (Hermon et al. 1996; Tabar et al. 1985). Besides the improvement of treatment (Burton et al. 2012), early detection of breast cancer has been suggested to be one of the most important contributors (Narod 2011; Smith et al. 2012).

Mammography (MAM) screening plays a central role in early detection of breast cancers in western countries. Multiple population-based randomized controlled trials (RCTs) of MAM screening have individually and collectively provided strong support for the efficacy of breast cancer screening (Gotzsche et al. 2011). Based on these RCTs and observational studies, different screening guidelines have been established due to different interpretations of the evidence (Tonelli et al. 2011). Although contentious academic debates exist over the balance of benefits and potential harms from MAM (No authors listed. 2011), mass screening of breast cancer has become a routine practice in many Western countries (US Preventive Services Task Force 2009; Smith et al. 2003).

Comparing with their western counterparts, Chinese women have a relatively lower risk of breast cancer. Chinese women tend to have small, dense breasts, which may reduce the sensitivity of MAM. Moreover, while the incidence of breast cancer shows a linear relationship with age in Western women, the peak incidence of the disease has been observed among Chinese women at ages of 45 and 49 years old (Leong et al. 2010). Therefore, the recommended MAM screening strategy in the West may not be practical in China. Recent years, ultrasonography (US), as a supplementary examination to MAM, has been suggested to improve the diagnostic performance of the imaging procedures in clinical practice, particularly among Asian women (Xiao et al. 2008). Evidence from community population, however, is lacking.

In this study, by taking advantage of the data from the Shanghai Society-based Breast Screening Program (SBSP) initiated in May 2008, a biennial mass screening of breast cancer designed based on recommended screening strategies in Western countries and the experience in clinical practice, we evaluated the performance of screening methods and screening modality, aiming at seeking an optimal breast cancer screening modality in Chinese women.

## Materials and methods

### Screening program and participants

**Figure 1:**
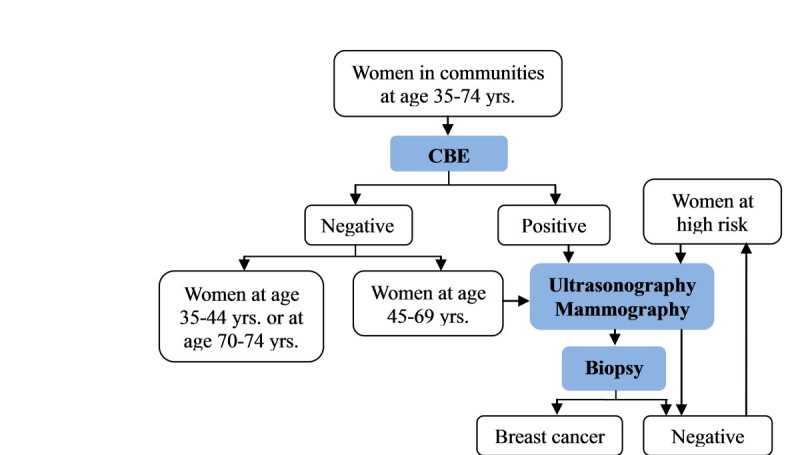
**Screening modality of breast cancer in the SBSP.**

This SBSP was conducted among Chinese women aged 35-74 years old in three consecutive stages. As shown in Figure 1, at first stage all eligible women living in Qibao county, Minhang district of Shanghai, were asked to be interviewed using a structured questionnaire and had a CBE after signing a consent form. The questionnaire included questions on demography characteristics, menstrual and reproductive factors, family history of breast cancer and history of any benign breast lesions. Women with positive CBE results and women at age of 45-69 years old, regardless of the CBE results, were preformed US and MAM examination in Cancer Hospital of Fudan University (second stage). Women either US positive or MAM positive had breast biopsy at the hospital (third stage). Breast cancer was diagnosed based on biopsy or pathological examination after surgery. Subjects with suspicious result of biopsy were recommended to take the next round of imaging examination.

During the period of May 2008 to September 2012, two rounds of breast cancer screening were conducted. Of the 14,464 women participating in the program, 2,117 (15%) only participated in the 2nd round of screening. A total of 64 subjects were detected as breast cancer in this program and 2 were identified by conducting record linkage with the Shanghai Cancer Registry System. This study was approved by the institutional ethics committee of Fudan University (Approval #: 20080460-5).

### Screening methods

CBE, MAM and US were used as screening methods in the SBSP. CBE was conducted by experienced surgeons in Qibao community hospital. Imaging screening was performed at the Breast Diagnostic Center in Cancer Hospital of Fudan University, which had staff facilities including surgeons, physicians, radiologists and pathologists. The MAM radiologists and the US operators completed respective imaging reports independently and were blinded to each other’s report.

In digital MAM (GE, 2000DTM (SD) or LORAD), exposure controls of MAM units were set at 25 kV with film density between 1.6 and 1.8D and daily calibration. Lateral image of each breast was obtained on two films (CC and MLO). The data were digitally stored and backed-up once a year. The films of MAM on high-resolution screen (BARCO) were read and interpreted independently by two radiologists following the Breast Imaging Reporting and Data System (BI-RADS) guidelines of the American College of Radiology (ACR).

The Doppler US images with high-frequency transducers at 7.5–10 MHz were obtained by two operators who did both the scanning and interpreting. The US operators scanned in vertical and horizontal parallel stripes covering the breast, axillary tail, and areola region, ensuring that each side of the entire breast volume was scanned twice. Axillary region was routinely scanned regardless of whether there was a significant abnormality in the ipsilateral breast. The daily workload of each US operator was restricted to fewer than 40 cases and average time per case was at least 5 min to guarantee the screening quality. Images of concerning lesions were taken specifically. The determination of a malignant lesion was based on ACR BI-RADS.

### Statistical analysis

All statistical analysis was performed using SAS software (version 9.2). We censored observational date on September 1, 2012. Thus, the years of exposure were computed from the date of examination (CBE or 2imaging screening) to the date of diagnosis or death, or the date of end. The sensitivity of a certain screening method was defined as the number of subjects correctly classified as breast cancers by this method divided by the number of all malignant cases diagnosed, and specificity was the ratio of true negatives by the method to all negatives. Positive predictive value (PPV) was calculated as the number of true positive divided by the total number of subjects who tested positive, and negative predictive value (NPV) referred to the proportion of subjects with a negative test result who were correctly diagnosed. *χ*^2^ test or Fisher’s exact test was used to compare the clinicopathological characteristics between screened and interval cancers, and diagnostic performance between different screening methods. Cox model was used to compute the hazard ratios (HR) and 95% confidential interval (CI) of risk factors of breast cancer. *P* < 0.05 was considered as statistically significant for two-sided tests.

## Results

### Incidence of breast cancer by characteristics of the participants

As presented in Table 1, the overall incidence of breast cancer was 194 per 100,000 person-years. The incidence was significantly higher in women with a family history, earlier age at menarche or older age at first live birth. Age, higher education and older age at menopause were also related to breast cancer risk, but the HRs did not reach significant.

**Table 1:** **Incidence of breast cancer by characteristics of the participants of the SBSP**

Characteristics	No. of subjects	No. of cases	Person years	Incidence (/100,000 PYs)	HR (95% CI)
All subjects	14464	66	34001	194	—
Age (yrs.)					
< 40	1020	0	2122	0	—
40-	2833	9	6848	131	0.60 (0.28, 1.27)
50-	5349	29	13200	220	1.00 (ref.)
60-	4213	23	9712	237	1.07 (0.62, 1.85)
70-	1049	5	2118	236	1.05 (0.40, 2.72)
Education					
Primary school or No formal education	3324	17	8940	190	1.23 (0.62, 2.44)
Junior middle school	5646	18	13361	135	1.00 (ref.)
Senior high school or technical school	3739	21	8099	259	1.86 (0.99, 3.49)
Junior college	1018	6	2148	279	2.44 (0.96, 6.19)
Regular college or above	737	4	1454	275	2.91 (0.97, 8.72)
Family history of breast cancer					
Yes	366	5	858	583	3.03 (1.22, 7.54)
No	14098	61	33143	184	1.00 (ref.)
Age at menarche (yrs.)					
≤ 13	2078	15	4582	327	1.99 (1.10, 3.58)
14-17	10330	44	24273	181	1.00 (ref.)
> 17	2053	7	5142	136	0.66 (0.29, 1.47)
Menopausal status					
Pre-menopause	4455	12	10510	114	0.81 (0.32, 2.04)
Post-menopause	9996	54	23462	230	1.00 (ref.)
Age at menopause (yrs.)					
< 45	721	4	1704	235	1.12 (0.41, 3.11)
45-54	8323	44	19523	225	1.00 (ref.)
≥ 55	952	6	2234	269	1.20 (0.51, 2.81)
Age at first live birth (yrs.)					
Nulliparity	299	0	643	0	—
< 24	3290	13	7940	164	0.99 (0.51, 1.92)
24-29	9121	39	21691	180	1.00 (ref.)
≥ 30	1750	14	3718	377	2.01 (1.09, 3.71)

Missing values were excluded from the analysis (3 subjects for age at menarche, 13 for menopause status and 4 for age at first live birth).

**Figure 2:**
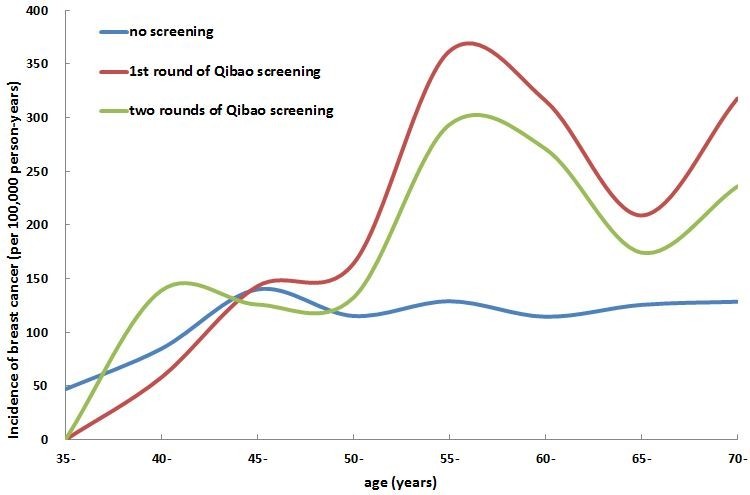
**Comparison of age-specific incidence of breast cancer between participants and non-participants of the SBSP.**

Figure 2 shows the age-specific incidence of breast cancer among the participants of the SBSP and the non-participants living in Minhang district whose incidence was from the Shanghai Cancer Registry system. A much higher breast cancer incidence was observed among participants of either the 1st round or the both rounds of screening than in non-participants. Interestingly, the peak incidence was observed among women around 45 years old in non-participants while the incidence increased with age among the participants, particularly for those participating in the 1st round of screening.

### Comparison of characteristics of detected and interval breast cancer cases

64 of 66 breast cancer cases were detected within two years of the last screening examination. As shown in Table 2, 51 cases were found during the 1st round of screening and 13 during the 2nd round of detecting. A total of 13 cases (20.3%) were interval cancer, namely, being diagnosed in two years but after three months of the imaging examinations. The percentage of interval cancer was higher in the age-group of 50-59 years, and among those with ER or PgR negative status, although the difference was not significant. The interval cancer also tended to be invasive ductal carcinomas and with large lump size.

**Table 2:** **Clinicopathological characteristics of breast cancer cases in the SBSP**

Characteristics	1st round screening		2nd round screening		Total	
	No. of cases	Interval cases (N, %)	No. of cases	Interval cases (N, %)	No. of cases	Interval cases (N, %)
Overall	51	11 (21.6)	13	2 (15.4)	64	13 (20.3)
Age group						
40-	6	0	3	0	9	0
50-	25	7 (28.0)	3	1 (33.3)	28	8 (28.6)
60-	16	3 (18.8)	6	1 (16.7)	22	4 (18.2)
70-	4	1 (25.0)	1	0	5	1 (20.0)
Pathological type						
DCIS	8	2 (25.0)	0	0	8	2 (25.0)
DCIS with micro-invasive	5	1 (20.0)	2	0	7	1 (14.3)
IDC	34	6 (17.7)	9	1 (11.1)	43	7 (16.3)
Special types	3	2 (66.7)	2	1 (50.0)	5	3 (60.0)
Size (mm)						
DCIS	13	3 (23.1)	3	0	16	3 (18.8)
< 20	12	3 (25.0)	5	2 (40.0)	17	5 (29.4)
20-	23	4 (17.4)	4	0	27	4 (14.8)
50-	2	1 (50.0)	0	0	2	1 (50.0)
Axillary lymph node status						
Negative	40	10 (25.0)	12	2 (16.7)	52	12 (23.1)
Positive	10	1 (10.0)	0	0	10	1 (10.0)
Stage						
DCIS ^a^	13	3 (23.1)	3	0	16	3 (18.8)
I	15	3 (20.0)	8	2 (25.0)	23	5 (21.7)
IIA	14	3 (21.4)	1	0	15	3 (20.0)
IIB	6	1 (16.7)	0	0	6	1 (16.7)
III-IV	2	1 (50.0)	0	0	2	1 (50.0)
ER status						
Positive	35	7 (20.0)	9	2 (22.2)	44	9 (20.5)
Negative	13	4 (30.8)	3	0	16	4 (25.0)
PgR status						
Positive	30	4 (13.3)	9	2 (22.2)	39	6 (15.4)
Negative	18	7 (38.9)	3	0	21	7 (33.3)

^a^ both DCIS and DCIS with micro-invasive;

Missing values were excluded from the analysis (1 for pathological type, 2 for tumor size, axillary lymph node status and stage, 4 for ER and PgR status).

Abbreviations: *DCIS* ductal carcinoma *in situ*, *IDC* invasive ductal carcinoma, *ER* estrogen receptor, *PgR* progesterone receptor.

### Performances of screening methods and modality

Among 14,464 participants, 13,906 subjects had CBE. The sensitivity of CBE alone was 61.4%, slightly higher than that of the US alone (53.7%). However, CBE showed quite lower specificity (51.5%) and PPV (0.5%) than US (98.5% and 17.4%, respectively). MAM had higher sensitivity (67.3%) and PPV (17.9%) than did US alone. When combining US and MAM in screening, the sensitivity increased to 79.3% (Table 3).

**Table 3:** **Overall screening performance of screening methods in the SBSP**

Screening methods	No. of tests	No. of patients	Sensitivity (%)	Specificity (%)	PPV (%)	NPV (%)
CBE	13906	57	61.4	51.5	0.5	99.7
US	9261	54	53.7	98.5	17.4	99.7
MAM	9238	55	67.3	98.1	17.9	99.8
US + MAM	9226	53	79.3	96.9	12.8	99.9
Screening modality ^a^	9994	50	76.0	97.2	12.1	99.9
Real practice of the modality	13906	57	75.4	81.3	1.6	99.9

^a^ Among women following the Qibao screening modality strictly.

Abbreviations: *CBE*: clinical breast examination; *US*: ultrasonography; *MAM*: mammography.

The three-stage Qibao modality was not conducted strictly in practice. About 30% of participants who should be subjected to imaging examination lacked US or MAM imaging result, and about 300 participants had unnecessary imaging examinations according to the screening strategy. The changes have resulted in a slightly lower sensitivity (75.4%) than the modality (76.0%), which was calculated among 9,994 women following the Qibao screening modality strictly.

Table 4 presents the performance of US and MAM screening by age groups. US alone had highest sensitivity in the youngest group (75%) and lowest one in the oldest group (50%). Conversely, MAM alone performed best in the oldest group and had lowest sensitivity in the youngest group (50.0%). Combined use of the two imaging examinations improved sensitivity in almost all age groups and increased the sensitivity as high as 85.7% in the group of 60-69 years old.

**Table 4:** **Performance of US and MAM screening by age groups in the SBSP**

Screening methods	No. of tests	No. of patients	Sensitivity (%)	PPV (%)
US				
Age group				
< 45	651	4	75.0	25.0
45-59	5261	25	52.0	14.0
60-69	3128	21	52.4	20.4
≥70	221	4	50.0	25.0
MAM				
Age group				
< 45	644	4	50.0	18.2
45-59	5255	26	65.4	13.4
60-69	3119	21	66.8	22.6
≥70	220	4	100.0	57.1
US + MAM				
Age group				
< 45	644	4	75.0	15.0
45-59	5245	24	70.8	8.7
60-69	3117	21	85.7	17.7
≥70	220	4	100.0	33.3

Abbreviations: *US* ultrasonography, *MAM* mammography, *PPV* positive predictive value.

As presented in Table 5, among 60 breast cancer cases having ER and PgR status data, the sensitivity of US alone was comparable between ER or PgR positive and negative patients. MAM, on the other hand, had a higher sensitivity among ER or PgR positive patients. The difference between PgR positive and negative patients reached significant (*P* = 0.02). The combined use of the two imaging examinations improved the sensitivity of screening regardless of ER or PgR status, but had a higher sensitivity in hormone receptor (ER and PgR) positive cancers than in those negative (90.0% vs. 63.2%, *P* = 0.03).

**Table 5:** **Sensitivity of US and MAM screening by ER or PgR status in the SBSP**

Screening methods	ER status			PgR status			ER and PgR status		
	Positive	Negative	***P*** value	Positive	Negative	***P*** value	Both positive	Either negative	***P*** value
US	52.8% (19/36)	50.0% (7/14)	0.86	51.6% (16/31)	52.6% (10/19)	0.94	53.3% (16/30)	50.0% (10/20)	0.82
MAM	72.2% (26/36)	53.3% (8/15)	0.19	78.1% (25/32)	47.4% (9/19)	0.02	77.4% (24/31)	50.0% (10/20)	0.04
US + MAM	82.9% (29/35)	71.4% (10/14)	0.44	90.3% (28/31)	61.1% (11/18)	0.03	90.0% (27/30)	63.2% (12/19)	0.03

Abbreviations: *ER* estrogen receptor, *PgR* progesterone receptor, *US* ultrasonography, *MAM* mammography.

### Potential adjustment of the Qibao screening modality

Based on the age-specific sensitivity of the screening methods in this population, we adjusted the screening modality to improve the effectiveness of screening or minimize the workload. If the age-group of the women having CBE, US and MAM was adjusted from 45-69 to 50-69 years old, the sensitivity would remain unchanged but the costs for 334 times of MAM and US could be saved. Alternatively, if all women over 70 years old with CBE positive results had MAM alone instead of having both MAM and US, the costs for 107 times of US could be saved without loss of effectiveness.

## Discussion

Based on recommended screening strategies in Western countries (No authors listed 2009; Smith et al. 2003) and screening experiences in other countries (Shetty 2011; Uchida et al. 2008), our current population-based breast cancer screening program conducted in Qibao county of Minhang district in Shanghai, China, was designed to combined use of MAM and US to early detect breast cancer in Chinese women. Considering the large population and limited resources in China, clinical breast examination (CBE) was also used as one of screening methods, which has been suggested as a good candidate for early detecting of breast cancer in developing countries like India and China (Jatoi 2003).

After two rounds of biennial screening, the incidence of breast cancer was much higher in participants than in non-participants. It also appears increase dramatically compared with the age-specific incidence rates in general female population of Shanghai, China (Shanghai Municipal Center for Disease Control and Prevention 2010; Shanghai Municipal Center for Disease Control and Prevention 2011). The incidence pattern by age-groups was greatly changed by the screening program. In non-participants, the incidence remained stable along age with a small peak around 45 years old, which is similar to the patterns observed in Asian women (Lee et al. 2009; Tonelli et al. 2011). In the participants, conversely, the incidence increased with increasing age, which is very close to the patterns in western countries (Lee et al. 2009). Early detection of breast cancer, particularly the 1st round of screening, may be the main reason for the change. That is, some patients who would be diagnosed naturally later were detected ahead, resulting in a temporally elevated incidence rate. However, due to that 2.5% of participants in our population had family history of breast cancer, much higher than 1.4% in the general population of Shanghai (DeRoo et al. 2010), selection bias could not be excluded.

Many factors may influence the accuracy of the screening methods. The performance of CBE mainly depends on the operator’s skills and experience. In this program, the CBE was conducted by several skilled and experienced surgeons, making the overall sensitivity as high as 61.4%. However, the specificity and PPV of CBE were quite lower than other screening methods. MAM alone shows a lower sensitivity in this population than it did in other ethnic populations (Mushlin et al. 1998), possibly due to smaller and denser breasts in Asian women. However, we find that, along with increasing age the sensitivity of MAM increased while that of US decreased, as a result combined use of the two methods greatly improved the overall diagnostic sensitivity, consistently with the findings in communities and in clinical practice (Ji et al. 2013). We also find that the hormone receptor status of breast cancer influence the sensitivity of MAM, but not the accuracy of US, providing further evidence for value of US as complementary tool to MAM in breast cancer screening among Chinese women.

The Qibao screening modality was designed to use different screening methods combinedly and focus on Chinese women possibly at high risk of the disease. Based on the age-specific incidence and age-specific sensitivity of different screening methods in this population, we find that the adjustment in age-group at high risk and the modification of screening methods can save costs for examination without trading off any effectiveness. These results suggest that the Qibao screening modality can be further improved. Cost-effective analysis of the program, however, is warranted to optimize and expand the use of the modality in China.

The strengths of the study included the representative sample of community female residents, large sample size and well-designed screening modality. However, only two rounds of screening have completed, limiting the statistical power in the analysis.

In conclusion, the Qibao modality is an effective strategy for breast cancer screening in Chinese women. The preliminary results of the first two rounds of screening provide implications on how to further improve the effectiveness of screening in Chinese population.

## Authors’ original submitted files for images

Below are the links to the authors’ original submitted files for images. Authors’ original file for figure 1 Authors’ original file for figure 2
