# Supplementary figures and images for: Performance of breast cancer screening methods and modality among Chinese women: a report from a society-based breast screening program (SBSP) in Shanghai
Source: Springerplus. 2013 Jun 24;2(1):276. doi: 10.1186/2193-1801-2-276 (PMC3724976; doi:10.1186/2193-1801-2-276)

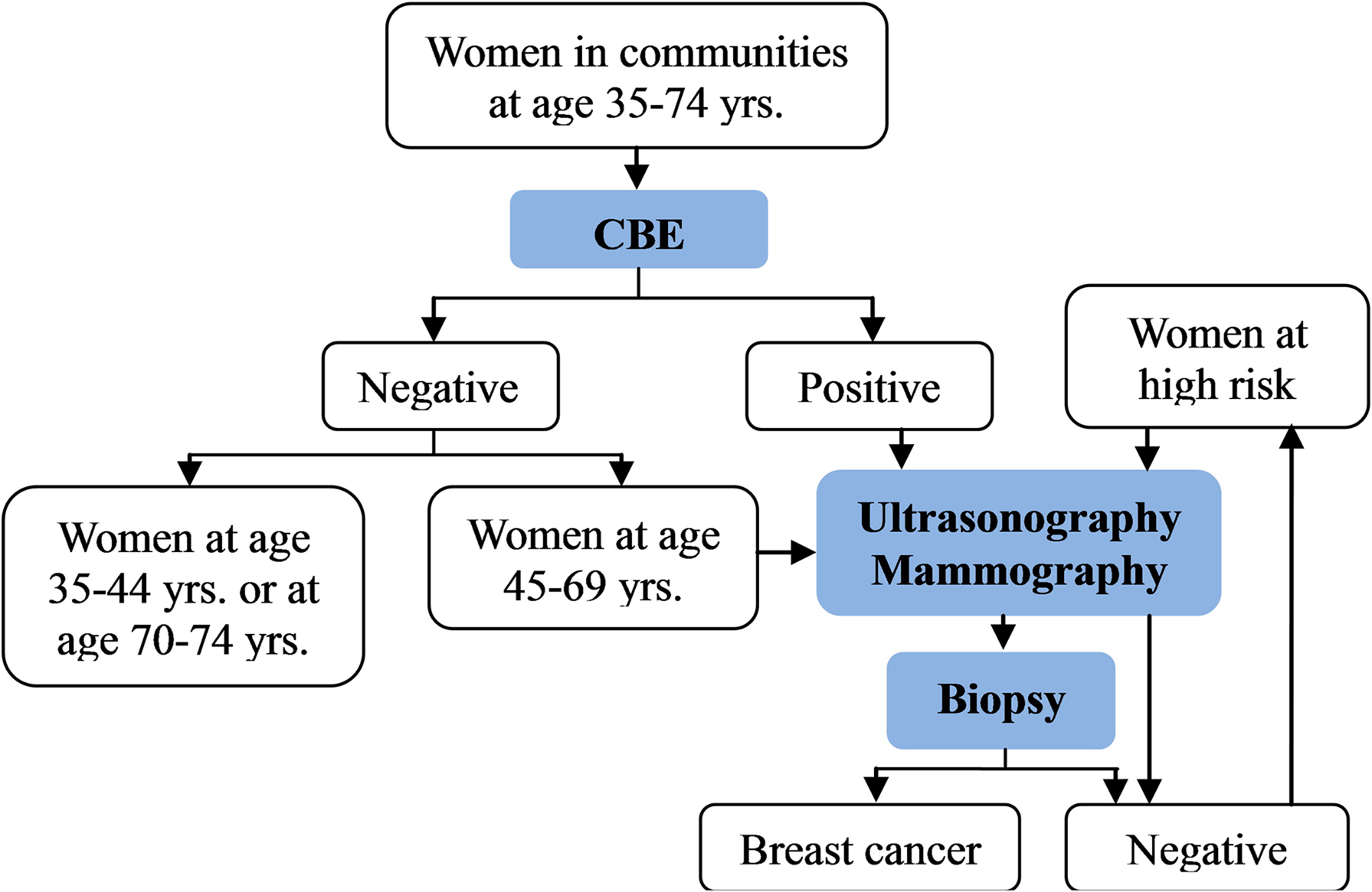

Supplement: Supplementary file 1 — Authors’ original file for figure 1 [file 40064_2013_364_MOESM1_ESM.tif]

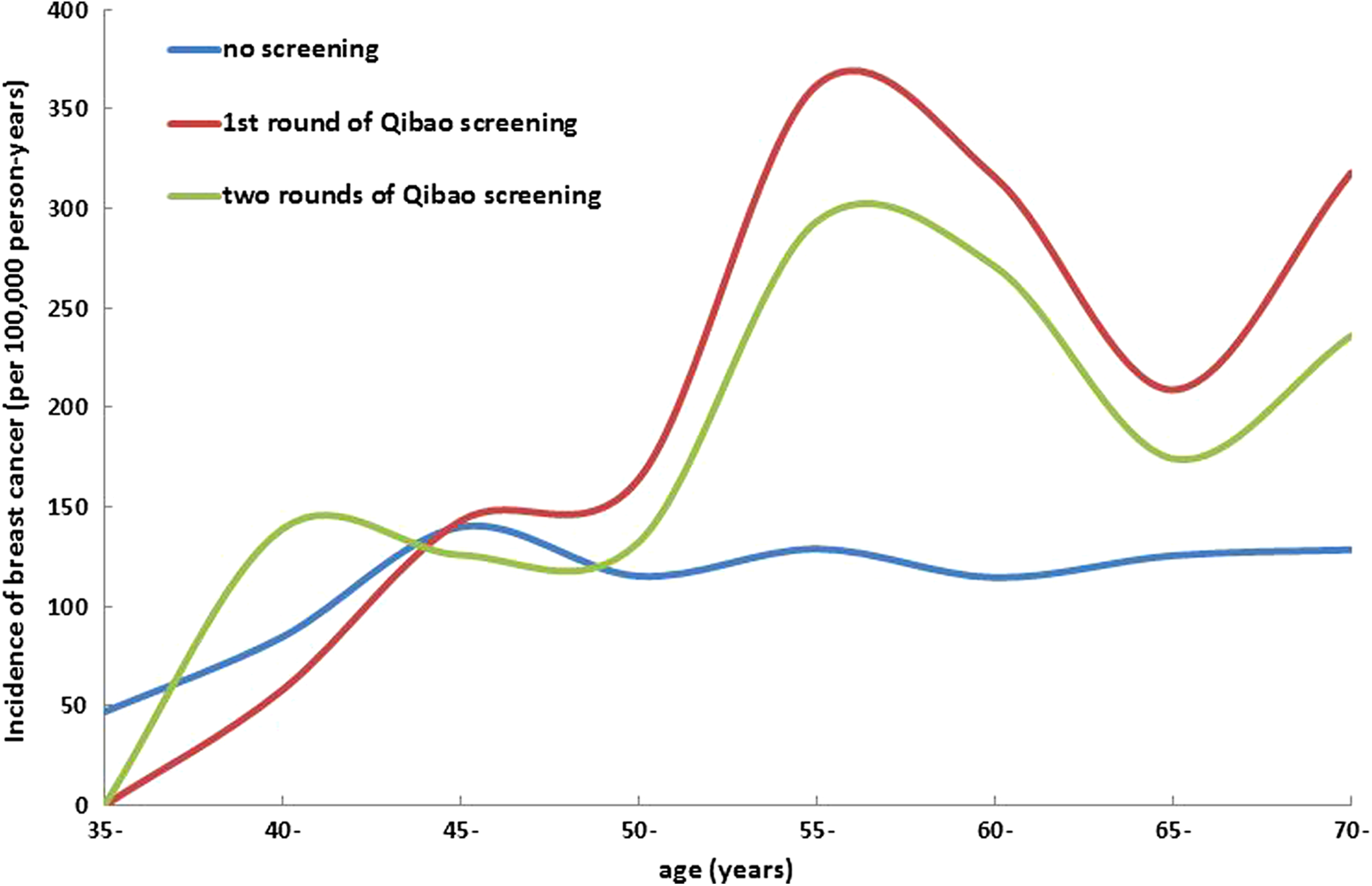

Supplement: Supplementary file 2 — Authors’ original file for figure 2 [file 40064_2013_364_MOESM2_ESM.tif]
